# Supplementary material for: The interactome of intact mitochondria by cross-linking mass spectrometry provides evidence for coexisting respiratory supercomplexes
Source: Mol Cell Proteomics. 2017 Dec 8;17(2):216–32. doi: 10.1074/mcp.RA117.000470 (PMC5795388; doi:10.1074/mcp.RA117.000470)
Supplement: Supplemental Data [file supp_RA117.000470_133922_0_supp_23978_fzffwf.zip › spectra_annotation/mito_spectra_annotation/120-1-13-1-4-1.pdf]

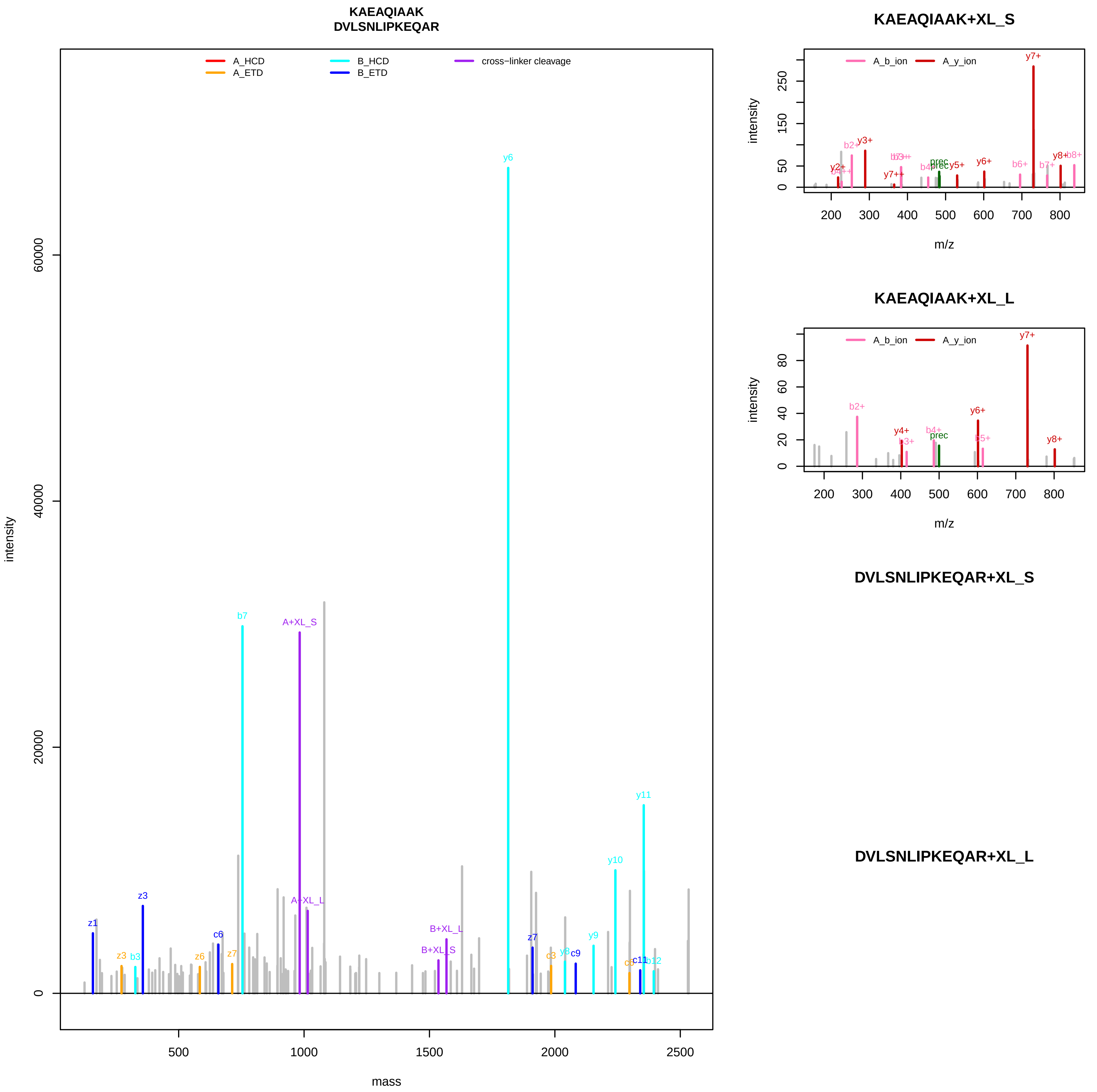
KAEAQIAAK
DVLSNLIPKEQAR
A_HCD
A_ETD
B_HCD
B_ETD
cross−linker cleavage
intensity
mass
60000
40000
20000
0
500
1000
1500
2000
2500
y6
b7
A+XL_S
A+XL_L
B+XL_L
B+XL_S
y11
y10
y9
z1
z3
z3
b3
c6
z6
z7
z7
c3
c9
c11
b12
KAEAQIAAK+XL_S
A_b_ion
A_y_ion
m/z
KAEAQIAAK+XL_L
DVLSNLIPKEQAR+XL_S
DVLSNLIPKEQAR+XL_L
